# Piperacillin–tazobactam as alternative to carbapenems for ICU patients

**DOI:** 10.1186/s13613-017-0334-x

**Published:** 2017-11-10

**Authors:** Benoit Pilmis, Vincent Jullien, Alexis Tabah, Jean-Ralph Zahar, Christian Brun-Buisson

**Affiliations:** 1 Service de maladies infectieuses et tropicales, Hôpital Necker Enfants malades, Service de maladies infectieuses et tropicales, Université Paris Descartes, Paris, France; 2 Equipe mobile de microbiologie clinique, Groupe Hospitalier Paris Saint-Joseph, Paris, France; 3 Service de Pharmacologie, Hôpital Européen Georges Pompidou, Université Paris Descartes, Paris, France; 4 INSERM U1129, Paris, France; 5 Intensive Care Unit, The Redcliffe Hospital, Brisbane, Australia; 6 Burns, Trauma and Critical Care Research Centre, The University of Queensland, Brisbane, Australia; 7 Département de Microbiologie Clinique, Unité de Contrôle et de Prévention du risque Infectieux, Groupe Hospitalier Paris Seine Saint-Denis, AP-HP, CHU Avicenne, 125 rue de Stalingrad, 9300 Bobigny, France; 8 Infection Control Unit, IAME, UMR 1137, Université Paris 13, Sorbonne Paris Cité, Paris, France; 9 Réanimation médicale, Hôpital Henri Mondor, Université Paris Est Créteil (UPEC), Créteil, France

**Keywords:** Carbapenems, ESBL, Alternatives, Ecological consequences, Outcome

## Abstract

Several studies suggest that alternatives to carbapenems, and particulary beta-lactam/beta-lactamase inhibitor combinations, can be used for therapy of extended-spectrum beta-lactamase-producing Enterobacteriaceae (ESBL-PE)-related infections in non-ICU patients. Little is known concerning ICU patients in whom achieving the desired plasmatic pharmacokinetic/pharmacodynamic (PK/PD) target may be difficult. Also, in vitro susceptibility to beta-lactamase inhibitors might not translate into clinical efficacy. We reviewed the recent clinical studies examining the use of BL/BLI as alternatives to carbapenems for therapy of bloodstream infection, PK/PD data and discuss potential ecological benefit from avoiding the use of carbapenems. With the lack of prospective randomized studies, treating ICU patients with ESBL-PE-related infections using piperacillin–tazobactam should be done with caution. Current data suggest that BL/BLI empirical use should be avoided for therapy of ESBL-PE-related infection. Also, definitive therapy should be reserved to patients in clinical stable condition, after microbial documentation and results of susceptibility tests. Optimization of administration and higher dosage should be used in order to reach pharmacological targets.

## Introduction

Since the 1980s, extended-spectrum beta-lactamase (ESBL)-producing Enterobacteriaceae (ESBL-PE) have been spreading worldwide [1, 2]. Several reports underline the concomitant increasing use of carbapenems [3, 4]. Indeed, the recently published 2015 ESAC report noted a threefold increased use of carbapenems between 2010 and 2014 [5]. This induces a selective pressure for carbapenem-resistant isolates, and recent data suggest that even a brief exposure to carbapenems increases the risk of colonization with carbapenem-resistant bacteria (CRB) in intensive care unit patients [6].

 To reduce the ecological risk associated with the increased consumption of last-line antibiotics, two main strategies are available: (1) searching for alternative treatments for ESBL-PE-related infections and (2) antimicrobial de-escalation (ADE). Therefore, the use of alternatives to carbapenems such as cephamycins, piperacillin–tazobactam and others for the treatment of ESBL-PE infections should be investigated. A recent systematic review, including two randomized controlled trials and 12 cohort studies, highlighted that the effects of ADE on antimicrobial resistance have not been properly studied [7]. However, this strategy is largely promoted by several scientific societies and specifically in critically ill patients [8, 9]. Indeed, for severely ill patients, international guidelines recommend the use of broad-spectrum antibiotics as first-line therapy to minimize the risk of inadequate initial antimicrobial treatment, and suggest streamlining initial antibiotic therapy and narrowing the spectrum whenever possible once the pathogen(s) are identified [10].

Until recently, the common rule is to treat infections caused by ESBL-producing organism with carbapenems. However, ESBLs are inhibited in vitro by beta-lactamase inhibitors and several studies have suggested the use of β-lactam/β-lactamase inhibitor combinations (BL/BLIs) such as piperacillin–tazobactam as a carbapenem-sparing strategy for the treatment of ESBL-PE-related infections [11–13]. The recent EUCAST and CLSI [14, 15] guidelines include BL/BLIs and other beta-lactams (cefepime, third generation cephalosporins, temocillin, cefoxitin) as treatment options for infections caused by ESBL-producing organisms. For a long time, AST categorization was based not only on MIC and zone diameter measurements but also on the detection of individual resistance mechanisms, i.e., interpretative reading. Even if in vitro results indicated susceptibility to a drug, the reported category was edited to “resistant” if the presence of a resistance mechanism was confirmed, e.g., in the case of extended-spectrum beta-lactamases (ESBLs). To limit the consumption of carbapenems, CLSI and EUCAST recently abandoned editing of AST reports based on the detection of ESBLs.

While several studies are conducted in ICU, data remain scarce concerning other beta-lactams in non-ICU- [16, 17] and ICU-infected [18–21] patients. Therefore, only BL/BLIs such as piperacillin–tazobactam (Pip–Taz) could be used in ICU patients, but there are concerns that: (1) no randomized controlled trials compared specifically carbapenems to Pip–Taz for the treatment of ESBL-PE-related infections [22]; (2) in vitro susceptibility to β-lactamase inhibitors might not predict clinical efficacy; and (3) the success of BL/BLIs depends on pharmacokinetic–pharmacodynamic target attainment, which current dosing recommendations may not guarantee. Therefore, alternatives are seldom used in clinical practice for treating serious infections caused by ESBL-PE.

In critically ill patients, pharmacokinetics of beta-lactam antibiotics differs from healthy volunteers. Lower than expected concentrations have been reported for meropenem, piperacillin, amoxicillin, as well as for cephalosporins [23–26]. Besides, the risk of treatment failure may be exacerbated when using antibiotics exposed to the inoculum effect [27], as are most beta-lactams.

This narrative review, based on microbiological, pharmacodynamics, clinical and ecological data, describes the available evidence for the use of Pip–Taz as an alternative to carbapenems in critically ill patients and to provide some guidance to prescribers for using these drugs when treating infections caused by ESBL-PE.

## Methods

### Literature search

A literature search was performed via PubMed, including all records from 1990 through April 2016. The following search pattern was applied: (ESBL OR extended-spectrum β-lactamases) AND (infection) AND (cefepime OR cefoxitine OR cephamycins OR flomoxef OR BL/BLI OR Piperacillin–tazobactam OR Carbapenems OR temocillin OR alternatives). Reference lists were cross-checked to identify further publications for possible inclusion. We restricted inclusion to studies published in the English, Spanish and French languages.

### Selection criteria

We screened and included studies in three categories according to the following criteria: (1) pharmacokinetics and pharmacodynamics studies, where all studies investigating the PK/PD of the potential alternatives to carbapenems in ICU patients were included. (2) For clinical studies, we restricted inclusion to studies reporting mortality of patients receiving empirical or definitive treatment with a non-carbapenem therapy for an ESBL bacteremia in adult patients. Patients with community-, hospital- and healthcare-associated bacteremia were eligible for inclusion. (3) Finally, considering ecological studies, we included any published article reporting carbapenem-resistant Enterobacteriaceae (CRE). Among the eligible articles, studies were included if they reported on exposure to any previous antibiotic class as a risk factor associated with CRE acquisition.

## Results

### Microbiological susceptibility

Several studies suggested that ESBL-PE were susceptible to non-carbapenem beta-lactams. However, the prevalence of susceptibility depends on the species concerned, the antibiotic class and local epidemiology. ESBL-producing *E. coli* is usually regarded as more susceptible to all beta-lactams than ESBL-producing *K. pneumoniae*, piperacillin–tazobactam (Pip–Taz) being the most effective antibiotic [28]. North American data from the 2010–2014 SMART programs find that 4, 10 and 46% of ESBL-producing *E. coli* were susceptible to ceftriaxone, cefepime and ceftazidime, respectively [28], whereas 96–98 and 69% of ESBL-producing *E. coli* isolates from urinary tract [29] and from patients with pneumonia [30] were found susceptible in vitro to Pip–Taz, respectively. Conversely, only 26.9% of ESBL-producing *Klebsiella* spp. isolates from patients with pneumonia were susceptible to Pip–Taz [30]. Asian data on ESBL-producing *E. coli* find similar susceptibilities, with 1.6, 9.5, 33.4 and 84.5% isolates susceptible to cefotaxime, cefepime, ceftazidime and Pip–Taz, respectively [29]. It is noteworthy that in silico PK/PD studies aiming to evaluate the use of alternatives to carbapenems for treatment of ESBL-PE infections suggest that ESBL-Kp susceptibility is overestimated by conventional methods in comparison with E-test susceptibility testing.

### Pharmacokinetics and pharmacodynamics studies

According to epidemiological data, two main antibiotics could be used as an alternative to carbapenems: piperacillin and cefoxitin. Others antibiotics suggested in the literature as temocillin, ceftolozane/tazobactam and/or ceftazidime/avibactam are less tested. Our goal was to define the optimal condition for using these antibiotics for ESBL-PE-related infections in ICU.

The pharmacokinetics of piperacillin in ICU patients was quite extensively investigated. There is, however, a lack of consensus on the pharmacokinetic/pharmacodynamic target to be achieved. Indeed targets as different as obtaining a free concentration > MIC (fT > MIC) or > 4 times the MIC (fT > 4xMIC) for 50 or 100% of a dose interval have been considered [31–36]. This is a crucial point as the dose to be administered will vary considerably according to the chosen target. There are, however, increasing data supporting a minimal efficacy criteria of fT > MIC = 100% in ICU patients, while a total trough concentration/MIC ratio of at least three was found to prevent the emergence of resistance in vitro [37–40]. Therefore, based on these more drastic PK/PD endpoints, it seems a dose of 4.5 g TID given as intermittent infusions should not be considered any more in ICU patients with normal renal functions [32, 36]. A 4.5-g × 4 daily dose appears more convenient, provided it is administered as prolonged infusion of at least 3 h [32, 34]. Indeed, for an intermittent bolus administration, a 4gx4 dose is associated with a very low probability of target attainment, even for the lowest PK/PD target of T > MIC = 50% [32]. However, even with a 4.5-g x 4 dose given by extended 3-h infusions, around one-third of the patients may not achieve a fT > MIC = 100%, which supports the need for an individual dose adjustment using therapeutic drug monitoring [35]. Such a result strongly supports the use of continuous infusion, and since this administration mode provides a better outcome than intermittent infusion [24], we believe a 16-g daily dose given as a continuous infusion, following a 4.5-g loading dose, should be considered as a starting point in ICU patients with normal renal function. Such an approach was found relevant for the treatment of ventilator-associated pneumonia, as it allowed the achievement of alveolar concentrations > 16 mg/L (i.e., the clinical breakpoint for gram-negative bacteria).

Slightly different results were observed in morbidly obese ICU patients, for whom the elimination half-life of piperacillin seems to be increased, compared to non-obese patients, resulting in an increased fT > MIC for equivalent doses [33]. Consequently, a 4.5-g × 4 daily dose given as a 4-h extended infusion should provide satisfying trough concentrations [33].

The pharmacokinetics of piperacillin in ICU patients undergoing continuous renal replacement therapy (CRRT) was also investigated, and similar results were found in case of venovenous hemofiltration or hemodiafiltration. A 4.5-g TID dose given as 30-min infusion should provide a free concentration > MIC for the entire dosing interval in almost all patients. Extending the infusion duration to 4 h should allow the attainment of several times the MIC. However, dose requirements seem to importantly depend on the membrane used and the effluent rate that are major aspects of CRRT poorly investigated to date [41, 42]. An interesting point is that piperacillin concentration in the dialysate effluent is equal to the free plasma concentration and can therefore be used for the individual adaptation of the dose via therapeutic drug monitoring (TDM) [43]. To our knowledge, the PK of piperacillin in the context of intermittent hemodialysis was not investigated to date in ICU patients. Based on the results obtained in sepsis-free volunteers with chronic renal failure [44], a dose of 4.5 g bid could be used as a starting point, with a subsequent TDM-guided individual adjustment of the dose. Conflicting results are available about the percentage of the dose that is eliminated by a 4-h session of hemodialysis (i.e., from 10 to 50%) [45, 46]. However, because a supplemental elimination is likely to occur during hemodialysis, it seems preferable to administrate the drug just after the end of the hemodialysis session.

Cefoxitin PK in ICU patients was not investigated to date. By using the PK parameters obtained in healthy subjects, it was shown that for a 8-g daily dose of cefoxitin, only an administration by continuous infusion provided a high probability to achieve targets of fT > MIC = 100% and fT > 4 × MIC = 100% for ESBL-PE [47]. However, since PK differences are expected in ICU subjects, PK data in this population are obviously needed [48].

Concerning temocillin, a 2-g TID dose given as intermittent 30-min infusion, provides a high probability to attain fT > MIC = 100% in ICU patients with normal renal function, provided the MIC is ≤ 4 mg/L. For higher MIC, administration of the same daily dose by continuous infusion is preferable [49]. In summary, among the different antibiotics suggested as alternatives to carbapenems, Pip–Tz is the one with the most frequent published PK/PD data in ICU. High daily doses and prolonged infusion should be promoted for ESBL-PE-related infections in ICU patients.

### Clinical studies

**Fig. 1:**
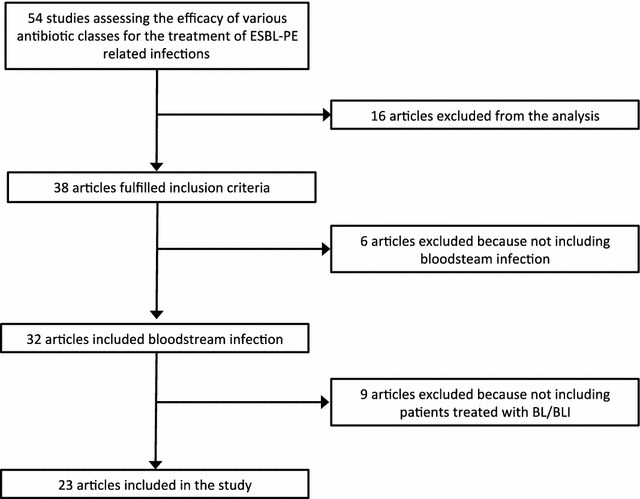
Flow diagram of the selection process of the included studies

The article selection process is shown in Fig. 1. Of the 54 articles selected initially, 23 provided data among patients treated with BL/BLIs for ESBL-producing Enterobacteriaceae-related infections (Table 1). Most of the published studies were retrospective (17/23; 73.9%), and all others were observational. Community-acquired, healthcare-associated and nosocomial infections were included without distinction. Among these 23 studies, 9 (39.1%), 6 (26%) and 7 (30.4%) evaluated antibiotic therapy as empirical therapy (ETC), definitive therapy or both, respectively. Among carbapenems, the selected molecule was available in 53% of included studies and imipenem–cilastatin was the most frequently used (45.7% of studies) followed by meropenem (35.2%) and ertapenem (19.1%).

**Table 1:** Studies characteristics

Author/year of publication	Study design, region	No. of patients, ESBL/total	Type of infection (*n*, %)	Bacteria (*n*, %)	ICU (*n*, %)	ETC/DTC	Treatment (*n*, %)	MIC	Administration (CI, PI or II)	Posology
Apisarnthanarak et al. [52]	SC case–control, 2003–2007, Thailand	36/146	UK (36, 100%)	*E. coli* *K. pneumoniae*	UK	ETC (36, 100%)	Cephalosporins (17, 47.2%) BL/BLI (10, 27.8%) Carbapenem (5, 13.9%) Fluoroquinolones (4, 11.1%)	N	UK	UK
Balakrishnan et al. [94]	MC retrospective cohort, 2008–2010, United Kingdom	42/42	UK	UK	UK	DTC (42, 100%)	Temocillin (42, 100%)	Y	II	Y
Bin et al. [95]	SC prospective cohort, 2002–2005, China	22/22	IIA (11, 50%) Primary bacteraemia (6, 27.2%) UTI (5, 22.7%)	*E. coli* (22, 100%)	UK	DTC (22, 100%)	Carbapenem (8, 36.4%) Cephalosporins (7, 31.8%) BL/BLI (7, 31.8%)	Y	Y	UK
Chaubey et al. [96]	MC prospective cohort, 2000–2007, Canada	79/79	Primary bacteraemia (39, 49.3%) UTI (38, 48.1%) Pneumonia (2, 2.5%) IIA (1, 1.3%)	*E. coli* (72, 91.1%) *K. pneumonia*e (7, 8.9%)	UK	ETC (74, 93.7%) DTC (79, 100%)	Carbapenem (16, 20.2%) BL/BLI (16, 20.2%) Aminoglycosides (16, 20.2%) Fluoroquinolones (16, 20.2%) Cephalosporins (16, 20.2%) Carbapenem (16, 20.2%) BL/BLI (16, 20.2%) Fluoroquinolones (16, 20.2%) Sulfamides (16, 20.2%) Aminoglycosides (16, 20.2%)	N	UK	UK
Chopra et al. [50]	MC retrospective cohort, 2005–2007, USA	145/145	UK	*E. coli* (24, 16.6%) *K. pneumoniae* (121, 83.4%)	Y (37, 25,5%)	ETC (128, 88.2%) DTC (110)	Cephalosporins (85, 58.6%) Carbapenem (50, 34.4%) Fluoroquinolones (6, 3.9%) Aminoglycosides (4, 3.1%) Carbapenem (103, 70.9%) Cephalosporins (41, 28.2%) BL/BLI (24, 16.4%) Fluoroquinolones (17, 11.8%) Amikacin (17, 11.8%) Tigecycline (12, 8.2%)	Y	UK	UK
Chung et al. [97]	SC retrospective cohort, 2005–2010, Taiwan	122/122	UTI (47, 38.5%) Primary bacteraemia (21, 17.2%) IIA (22, 18%) Pneumonia (6, 4.9%) CVC (6, 4.9%) Skin and soft tissue (4, 3.3%) Surgical site infection (3, 2.5%) Other (13, 10.7%)	*E. coli* (122, 100%)	UK	DTC (107 87.7%)	Carbapenem (71, 57.9%) Non-BL/BLI (48, 39.3%) BL/BLI (3, 2.8%)	N	UK	UK
De Rosa et al. [98]	SC retrospective cohort, 2000–2007, Italy	128/128	Primary bacteraemia (61, 47.6%) IIA (55, 43%) UTI (12, 9.4%)	*E. coli* (80, 62.5%) *K. pneumoniae* (28, 21.9%) *P. mirabilis* (20, 15.6%)	Y (8, 6.3%)	ETC (97 75.8%)	Carbapenem (101, 79.3%) BL/BLI (10, 8.2%) Fluoroquinolone (8, 6.2%) Trimethoprim/sulfamethoxazole (1, 1%) Aminoglycosides (8, 6.2%)	N	UK	UK
Du et al. [16]	SC retrospective cohort, 1997–1999, China	23/85	Primary bacteraemia (9, 39.1%) IIA (5, 21.7%) Pneumonia (4, 17.4%) UTI (2, 8.7%) CVC (2, 8.7%) Other (1, 4.4%)	*E. coli* (16, 69.5%) *K. pneumoniae* (7, 30.5%)	N	DTC (23, 100%)	Carbapenem (13, 56.5%) Cephalosporins (7, 30.4%) Fluoroquinolone (2, 8.7%) Aminoglycosides (1, 4.4%)	N	UK	UK
Endimiani et al. [99]	SC retrospective cohort, 1997–2004, Italy	9/23	Primary bacteraemia (5, 55.6%) UTI (4, 44.4%)	*P. mirabilis* (9, 100%)	UK	ETC (9, 100%) DTC (9, 100%)	Cephalosporins (5, 55.6%) BL/BLI (4, 44.4%) Cephalosporins (4, 44.4%) BL/BLI (3, 33.3%) Carbapenem (2, 22.3%)	Y	Y	UK
Ferrandez et al. [100]	Retrospective cohort, 2000–2006, Spain	53/53	UK	*E. coli* *K. pneumoniae*	UK	–	Carbapenem (30, 56.6%) BL/BLI (5, 9.4%) Fluoroquinolone (4, 7.5%) Cephalosporins (2, 3.8%) Other (12, 22.7%)	Y	UK	UK
Gudiol et al. [101]	SC prospective observational study, 2006–2008, Spain	17/135	Primary bacteraemia (9, 52.9%) IIA (6, 35.3%) UTI (1, 5.9%) Other (1, 5.9%)	*E. coli* (17, 100%)	Y (2, 12%)	ETC (17, 100%) DTC (17, 100%)	BL/BLI (6, 35.3%) Carbapenem (5, 29.4%) Cephalosporins (5, 29.4%) Monobactam (1, 5.9%) Carbapenem (14, 82.3%) BL/BLI (2, 11.8%) Fluoroquinolone (1, 5.9%)	N	UK	UK
Gutiérez-Gutiérez et al. [51]	MC, retrospective cohort study, 2004–2013, International	601/601	UTI (272, 45.2%) Other (258, 42.9%) IIA (71, 11.8%)	*E. coli* (439, 73%) *K. pneumoniae* (114, 19%) Other (48, 8%)	Y (64, 10.7%)	ETC (365, 60.7%) DTC (601, 100%)	Carbapenem (195, 53.5%) BL/BLI (169, 46.5%) Carbapenem (509, 84.7%) BL/BLI (92, 15.3%)	N	Y	II
Harris et al. [102]	SC retrospective cohort study, 2012–2013, China	92/92	UTI (43, 46.7%) Primary bacteramia (39, 42.2%) IIA (10, 11.1%)	*E. coli* (79, 85.9%) *K. pneumoniae* (13, 14.1%)	Y (11, 12.1%)	DTC (47, 51%)	Carbapenem (23, 48.9%) BL/BLI (24, 51.1%)	N	Y	II
Kang et al. [17]	SC retrospective cohort study, 1998–2002, South Korea	133/133	IIA (82, 61.6%) Primary bacteraemia (33, 24.8%) UTI (14, 10.5%) Pneumonia (4, 3.1%)	*E. coli* (67, 50.4%) *K. pneumoniae* (66, 49.6%)	N	ETC (133, 100%) DTC (133, 100%)	Non cephalosporins (29, 21.8%) Cephalosporins (104, 78.2%) Non cephalosporins (101, 75.9%) Cephalosporins (32, 24.1%)	Y	UK	UK
Kang et al. [69]	MC retrospective cohorts, 2008–2010, South Korea	114/114	UK	*E. coli* (78, 68.4%) *K. pneumonia* (36, 31.6%)	UK	ETC (114 100%)	Carbapenem (78, 68.4%) Piperacillin/tazobactam (36, 31.6%)	N	UK	UK
Lee et al. [53]	SC retrospective cohort, 2004–2005, Taiwan	27/27	Pneumonia (15, 55.5%) IIA (5, 18.5%) UTI (3, 11.1%) Primary bacteraemia (3, 11.1%) Other (1, 3.8%)	*K. pneumonia* (27, 100%)	Y (13, 48.1%)	DTC (27, 100%)	Carbapenem (20, 74%) Flomoxef (7, 26%)	Y	UK	UK
Lee and *al.* [21]	SC retrospective cohort, 2001–2008, Taiwan	121/206	CVC (48, 39.6%) Primary bacteraemia (32, 26.4%) Pneumonia (12, 9.9%) SSTI (9, 7.4%) UTI (9, 7.4%) IIA (6, 4.9%) Other (5, 4.3%)	*E. cloacae* (121, 100%)	Y (78, 64.4%)	ETC (114, 94.2%) DTC (114, 94.2%)	Cephalosporins (59, 49.1%) Carbapenem (26, 21%) BL/BLI (14, 11.4%) Other beta-lactam (13, 10.5%) Fluoroquinolones (3, 2.6%) Other Antibiotics (6, 5.4%) Carbapenem (53, 46.5%) Cephalosporins (38, 33.3%) Fluoroquinolones (16, 14%) BL/BLI (3, 2.6%) Other β-lactam (3, 2.6%) Other antibiotic therapy (1, 1%)	N	UK	UK
Lee et al. [54]	MC retrospective cohort, 2002–2007, Taiwan	178/178	Pneumonia (43, 24.1%) UTI (39, 21.9%) CVC (37, 20.8%) IIA (28, 15.7%) Primary bacteraemia (25, 14%) SSTI (11, 6.3%)	ND	UK	DTC (178, 100%)	Carbapenem (161, 90,4%) Cefepime (17, 9.6%)	Y	Y	II
Lee et al. [103]	MC retrospective cohort, 2007–2012, Taiwan	389/389	UTI (88, 22.6%) CVC (86, 22.1%) Pneumonia (80, 20.5%) IIA (61, 15.7%) Primary bacteraemia (62, 16%) SSTI (12, 3.1%)	*E. coli* (156, 40.1%) *K. pneumoniae* (233, 59.9%)	UK	DTC (389, 100%)	Carbapenem (257, 66%) Flomoxef (132, 34%)	Y	UK	UK
Matsumura et al. [104]	MC retrospective cohort, 2005–2014, Japan	113/1440	UTI (57, 50.4%) IIA (32, 28.3%) Primary bacteraemia (19, 16.8%) Other (5, 4.5%)	*E. coli* (113, 100%)	UK	ETC (71, 62.8%) DTC (113, 100%)	Carbapenem (45, 63.7%) Cefmetazole/flomoxef (26, 36.6%) Carbapenem (54, 47.8%) Cefmetazole/flomoxef (59, 52.2%)	Y	UK	UK
Ofer-Friedman et al. [20]	MC retrospective cohort, 2008–2012, International	79/79	Pneumonia (27, 34.2%) SSTI (22, 27.8%) IIA (20, 25.3%) Primary bacteraemia (6, 7.6%) Undetermined (4, 5.1%)	*E. coli* (42, 53.1%) *K. pneumoniae* (22, 27.8%) *P. mirabilis* (15, 19.1%)	> 50%	ETC (33, 41.8%) DTC (79, 100%)	Carbapenem (24, 72.7%) Piperacillin/tazobactam (9, 27.3%) Carbapenem (69, 87.3%) Piperacillin/tazobactam (10, 12.7%)	Y	UK	UK
Qureshi et al. [105]	MC retrospective cohort, 2005–2008, USA	21/UK	UK	*E. cloacae* (21, 100%)	UK	ETC (21, 100%)	Cephalosporins (9, 42.8%) Carbapenem (8, 38%) BL/BLI (4, 19.2%)	Y	UK	UK
Paterson et al. [55]	Post hoc analysis MC prospective cohort, 1996–1997, International	85/455	UK	*K. pneumoniae* (85, 100%)	UK	ETC (71, 83.5%)	Monotherapy Carbapenem (27, 38%) Fluoroquinolones (11, 15.5%) Cephalosporins (5, 7%) BL/BLI (4, 5.6%) Aminoglycosides (2, 2.8%) Combination therapy (15, 21.1%) Sequential monotherapy (7, 10%)	N	UK	UK
Pilmis et al. [106]	MC retrospective cohort, 2011, France	13/13	Primary bacteraemia (11, 84.6%) UTI (2, 15.4%)	*E. coli* (5, 38.4%) *K. pneumoniae* (7, 53.8%) *E. cloacae* (1, 7.8%)	UK	ETC (13, 100%) DTC (13, 100%)	Carbapenem (12, 92.3%) Cefoxitin (1, 7.7%) Carbapenem (11, 84.6%) Cefoxitin (2, 15.4%)	N	UK	UK
Retamar et al. [107]	Post hoc analysis MC prospective cohort, 2001–2007, Spain	39/39	UTI (11, 28.2%) Other source (28, 71.8%)	*E. coli* (39, 100%)	UK	ETC (39, 100%)	BL/BLI (39, 100%)	Y	UK	UK
Rodriguez-Bano et al. [12]	Post hoc analysis MC prospective cohorts, 2001–2007, Spain	192/192	UTI or IIA (121, 63%) Other sources (71, 37%)	*E. coli* (192, 100%)	Y (24, 12.6%)	ETC (103, 53.6%) DTC (174, 90.6%)	Carbapenem (31, 30%) BL/BLI (72, 70%) Carbapenem (120, 68.9%) BL/BLI (53, 31.1%)	N	UK	UK
Tamma et al. [19]	MC, Prospective cohort, 2008–2015, USA	213/331	CVC (97, 45.5%) IIA (55, 25.8%) UTI (44, 20.6%) Pneumonia (17, 8.1%)	*K. pneumoniae* (145, 68%) *E. coli* (66, 31%) *P. mirabilis* (2, 1%)	Y (71, 33.3%)	ETC (213, 100%)	Carbapenem (110, 51.6%) BL/BLI (103, 48.4%)	Y	UK	UK
Tsai et al. [108]	MC retrospective cohort, 2005–2012, Taiwan	47/47	UTI (24, 51%) Pneumonia (9, 19.1%) SSTI (7, 14.9%) CVC (5, 10.6%) IIA (3, 6.4%) Primary bacteraemia (2, 4.3%)	*P. mirabilis* (47, 100%)	UK	DTC (40, 85.1%)	Carbapenem (21, 52.5%) BL/BLI (13, 32.5%) Other antibiotic therapy (6, 15%)	Y	UK	UK
Tumbarello et al. [109]	SC retrospective cohort, 1999–2004, Italy	186/186	Primary bacteraemia (86, 46.2%) UTI (53, 28.4%) IIA (24, 12.9%) SSTI (20, 10.7%) Pneumonia (6, 3.2%) CVC (5, 2.7%)	*E. coli* (104, 55.9%) *K. pneumoniae* (58, 31.2%) *P. mirabilis* (24, 12.9%)	UK	ETC (186 100%) DTC (171, 91.9%)	BL/BLI (45, 24.2%) Fluoroquinolones (45, 24.2%) Cephalosporins (38, 20.9%) Carbapenems (29, 15.4%) Aminoglycosides (29, 15.4%) Carbapenems (61, 35.7%) BL/BLI (55, 32.2%) Aminoglycosides (30, 17.5%) Fluoroquinolones (25, 14.6%)	Y	UK	UK
Tuon et al. [110]	SC retrospective cohort, 2006–2009, Brazil	28/58	UK	*E. cloacae* (28, 100%)	UK	DTC (25, 89.2%)	Carbapenems (15, 60%) BL/BLI (4, 16%) Non-BL/BLI (6, 24%)	N	UK	UK
Tuon et al. [90]	SC retrospective cohort, 2006–2009, Brazil	63/104	UK	*K. pneumoniae* (63, 100%)	UK	DTC (62, 98.4%)	Carbapenems (43, 69.3%) Non-BL/BLI (17, 27.4%) BL/BLI (2, 3.1%)	N	UK	UK
Wang et al. [95]	MC, prospective cohort, 2006–2015, USA	68/68	CVC (30, 44.1%) UTI (21, 30.9%) IIA (15, 22.1%) Pneumonia (10, 14.7%) SSTI (2, 2.9%)	*Klebsiella sp.* (42, 62%) *E. coli* (24, 34%) *P. mirabilis* (2, 3%)	Y (20, 29%)	ETC (68, 100%)	Carbapenem (51, 75%) Cephalosporins (17, 25%)	N	UK	UK

*BL/BLI* beta-lactam/beta-lactamase inhibitor, *CVC* central venous catheter, *DTC* definitive therapy cohort, *ETC* empirical therapy cohort, *ICU* intensive care unit, *IIA* intra-abdominal infection, *MC* multicentric, *SC* single center, *SSTI* skin and soft tissue infection, *UK* unknown, *UTI* urinary tract infection

Only 3 (13%) studies reported the doses of antibiotics [19, 20, 50] and none reported the modalities of antibiotic’s administration. Indeed administered doses in patients without renal failure were variable; however, imipenem was used in most cases at an average dose of 0.5 g every 6 h, whereas 1 g every 8 h and 1 g every 24 h were used for meropenem and ertapenem, respectively. The two species most frequently involved were *E. coli* and *K. pneumoniae.* All patients included in these studies had bacteremia, and the two most frequent sites of infection were urinary tract and intra-abdominal infection. MIC was taken into account in adjusting antibiotic therapy in 11 (47.8%) of the 23 studies.

As mentioned above, 11 studies included between 6 and 131 ICU patients. In fact, some of the same patients were included in different cohorts [12, 51]. Only 4 studies [52–55] included patients with pneumonia caused by ESBL-PE, representing 8–50% of patients with ESBL-PE-related infections, indicating that less than 30 patients with ESBL-related pneumonia could be evaluated. Data regarding outcome for patients treated with carbapenems versus alternatives were available from 20 (86.9%) of the 23 studies including bacteremic patients. Surprisingly, potential confounding factors, such as severity of underlying diseases or of infection, were seldom reported.

Among studies including ICU patients, 6 (56%) compared BL/BLIs to carbapenems as empirical therapy. However, BL/BLIs was the only alternative compared to carbapenems in only 3 studies [12, 20, 51]. In these studies, *E. coli* and *K. pneumoniae* represented more than two-third of the isolates and MICs were taken into account in only one study [20]. The difference of mortality didn’t reach statistical significance in two studies [12, 51]. However, Ofer-friedman et al. [20] conducted a multicenter observational study including non-urinary BSI and comparing BL/BLI to carbapenem for the treatment of ESBL. In contrast to other studies, E*. coli* accounted for only half of the bloodstream infections; the median piperacillin MIC was 8 mg/L, and approximately half of patients required ICU care. In this study, the mortality was significantly higher in the piperacillin–tazobactam group [OR 7.9 (1.2–53)]. Thus, BL/BLIs may lead a poorer outcome than carbapenem therapy for critically ill patients with ESBL-PE infection from non-urinary sources.

Finally 7/11 (63%) studies compared BL/BLIs to carbapenems as definitive therapy, of which 4 (36.3%) compared BL/BLIs as the only alternative to carbapenems [12, 19, 20, 22, 51]. It should be noted that only one of these studies took into account MICs [20], whereas none took into account dosages and modalities of administration for assessing the effectiveness of therapy [19].

### Ecological studies

While initial research suggested the relative safety of imipenem–cilastatin on the intestinal microbiota [56], the recent analysis of rectal colonization of large number of ICU patients found that even a brief exposure to imipenem is a risk factor for carriage of resistant GNB in the intestinal flora [6].

The effect of non-carbapenem antibiotics on the emergence of multidrug-resistant bacteria and specifically carbapenem resistance is a major issue. In animal models, imipenem–cilastatin had no effect on the indigenous microflora [56]. In a mouse model, clindamycin and piperacillin–tazobactam promoted colonization, while ertapenem did not promote the establishment of intestinal colonization with KPC-Kp [57]. Also several authors highlighted the risk associated with the emergence/selection of resistant strains when using Pip–Taz. Firstly, in vitro/in vivo studies [27] suggested that Pip–Taz seems to be less resistant to the inoculum effect comparing to carbapenems. Secondly, several clinical studies [58] underlined the risk of promoting vancomycin-resistant enterococci (VRE) colonization. Finally the emergence of carbapenem-resistant PE has been documented for a variety of antibiotics in the clinical setting [59] (Table 2): Fluoroquinolones [60, 61], extended-spectrum cephalosporin [62], antipseudomonal penicillins [63] and β-lactams/β-lactamase inhibitors [64] have all been identified as risk factors for carbapenem resistance in *Klebsiella pneumoniae*.

**Table 2:** Studies addressing the risk related to previous antibiotic therapy and emergence of carbapenem-resistant Enterobacteriaceae

	Year	Study design	Type of infection	Antibiotic concerned	OR, 95 % CI
Wang [62]	2016	Retrospective case–case–control	Nosocomial infection	Third–fourth-generation cephalosporins Carbapenems	4.557 (1.971–10.539) 4.058 (1.753–9.397)
Mittal G [80]	2016	Prospective	Colonization	Aminoglycosides	4.14 (1.14–14.99)
Ling [81]	2015	Retrospective case–control	Infection or colonization	Penicillins Glycopeptides	4.640 (1.529–14.079) 5.162 (1.377–19.346)
Jiao Y [82]	2015	Retrospective case–control	Infection or colonization	Glycopeptides Cefoperazone plus sulbactam	43.84 (1.73–1111.9) 49.56, (1.42–1726.72)
Candevir [83]	2015	Retrospective cohort	Infection	Meropenem Third-generation cephalosporins	3.244 (1.193–8.819) 3.590 (1.056–12.209)
Gómez Rueda [84]	2014	Retrospective case–case–control	Infection	Carbapenems	3.3 (1.2–9.3)
Ahn [85]	2014	Retrospective case–control	Colonization/infection	Fluoroquinolones Carbapenems	2.82 (1.14–6.99) 4.56 (1.44–14.46)
Mantzarlis [86]	2013	Prospective cohort	Pneumonia	Colistin*	1.156 per day (1.010–1.312)
Dizbay [87]	2013	Prospective cohort	Nosocomial infection	Imipenem	3.35 (1.675–6.726)
Orsi [88]	2013	Retrospective case control	BSI	Carbapenem	7.74 (1.70–35.2)
Chang [89]	2011	Retrospective case–control	BSI	Carbapenem	29.17 (1.76–484.70)
Falagas [63]	2007	Retrospective case control	KPC infection	Fluoroquinolones Antipseudomonal antibiotics	4.54 (1.18–11.54) 2.6 (1.00–6.71)
Schwaber [61]	2008	Retrospective case–case–control	CRKp colonization	Antibiotics Fluoroquinolones	4.4 (1–19.2) 7.2 (1.1–49.4)
Gasink [60]	2009	Retrospective case–control	KPC infection/colonization	Fluoroquinolones Third-generation cephalosporin	3.39 (1.5–7.66) 2.55 (1.18–5.22)
Papadimitriou [64]	2012	Prospective cohort	CRKp colonization	BL/BLI Carbapenems	6.7 (1–26.2) 5.2 (1–32.9)
Tuon [90]	2012	Retrospective case–control	KPC bacteremia	Fluoroquinolones	28.9 (1.85–454.6)
Papadimitriou [91]	2014	Prospective cohort	KPC bacteremia	Aminoglycosides	2.3 (1.1–4.7)
Gagliotti [79]	2014	Case–control	KPC colonization	Carbapenems Any antibiotic (other than carbapenems)	3.67 (1.37–9.83) 2.83 (1.10–7.31)
Maseda [92]	2016	Retrospective	CPE isolate colonization	Third–fourth-generation cephalosporins BL/BLI	27.96 (6.88–113.58) 11.71 (4.51–30.43)

*KPC Klebsiella pneumoniae*-producing carbapenemase, *CRKp* carbapenem-resistant *Klebsiella pneumoniae*, *BL/BLI* beta-lactams associated with beta-lactamase inhibitors, *BSI* bloodstream infection

Whether carbapenem is the only antibiotic class associated with the selection of carbapenem-resistant gram-negative isolates is an important issue, especially regarding the worldwide spread of carbapenemase-producing Enterobacteriaceae (CPE). These data suggest that antibiotics that disturb the intestinal anaerobic microflora and lack significant activity against KPC-Kp may promote colonization by this organism [65] (Table 3).

**Table 3:** (Adapted from [14, 15]) usual breakpoints and susceptibility of ESBL-producing Enterobacteriaceae

	Susceptibility (%)	Breakpoints (mg/L)	Ecological impact	Comments
Third-generation cephalosporins	*Escherichia coli*: < 10% *Klebsiella* species: 3%	EUCAST: S ≤ 1 CLSI: S ≤ 1	+++	Only for targeted therapy or de-escalation MIC required
Cefepime	*E. coli*: 5–30% *K. pneumoniae*: 5–60%	EUCAST: S < 1 CLSI: S ≤ 2	+++	Frequent failure if MICs > 1 mg/L MIC required
Cefoxitin	*E. coli*: 80%	EUCAST: NA	++	PK optimization
Ceftolozane–tazobactam	*E. coli*: 85–95% *K. pneumoniae*: 40–65%	EUCAST: S ≤ 1 CLSI: S ≤ 8	?	
Ceftazidime–avibactam	*E. coli*: 98–100% *K. pneumoniae:* 90–100%	EUCAST: S ≤ 8 CLSI: S ≤ 8	?	Probably as effective as carbapenems
Temocillin	*E. coli* 61% (CMI ≤ 8) *E. coli* 99% (CMI ≤ 32)	EUCAST: S ≤ 8 EUCAST: S ≤ 32 (urinary) CLSI: S ≤ 8 CLSI: S ≤ 32 (urinary)	±	PK optimization (high dosage and prolonged infusion)

*CLSI* Clinical and Laboratory Standard Institute, *EUCAST* European Committee on Antimicrobial Susceptibility Testing, *MIC* minimum inhibitory concentration, *NA* not applicable, *PK* pharmacokinetic, *VAP* ventilator-associated pneumonia

## Discussion and conclusion

In our review of BL/BLI for the treatment of ESBL-PE, we found that they may be an alternative to carbapenems in a selected number of cases, based on antibiogram and CMI data, and always with pk/pd optimization.

The use of alternatives for empirical therapy in suspected ESBL-related infections is usually limited by the level of resistance [66], the risk of selecting resistant mutants [6] and clinical effectiveness [19]. We focused on BL/BLIs, chiefly piperacillin–tazobactam. Indeed, based on epidemiological data, the use of third- or fourth-generation cephalosporins such as cefepime is limited because of a high proportion of resistant isolates [66] that varies between 10 and 50% and concerns on their clinical efficacy with the associated risks of adverse patient outcomes [54]. The use of other alternatives such as temocillin is limited by unfavorable PK/PD parameters in critically ill patients [49].

The efficacy of Pip/Taz antibiotics on ESBL-PE depends on the variety and amount of enzyme produced by the isolates (Table 4). Overall, the rate of susceptibility of ESBL-PE to Pip/Taz is around 80% [28]. It may be reduced when the organisms produce multiple ESBLs, particularly if they also harbor an AmpC beta-lactamase [67]. Also it will vary within and between beta-lactamases classes [68]. The presence of additional resistance mechanisms may further decrease the activity of Pip/Taz against ESBL-producing organisms.

**Table 4:** (Adapted from Bonomo and Van Duin) Activity in clinical practice of different beta-lactamase inhibitors, according to type of enzymes [68, 111]

Enzymes	Class	Substrates	Clavulanic acid	Sulbactam	Tazobactam	Avibactam
TEM-1, TEM-2, SHV-1	A	Penicillins, early cephalosporins	+	–	+	+
TEM-3, SHV-2 CTX-M-14	A	Extended-spectrum cephalosporins, monobactams	–	–	+	+
KPC-2, KPC-3	A	Broad spectrum including carbapenems	–	–	–	+
IMP-1, NDM-1, VIM-1	B	Broad spectrum including carbapenems, but not monobactams	–	–	–	–
Escherichia coli AmpC	C	Cephalosporins	–	–	±	+
OXA-48	D	Carbapenem	–	–	–	+

In the absence of a well-designed prospective randomized study comparing carbapenems to non-carbapenems in ICU patients infected with ESBL-PE, we must rely on the evidence provided by observational data. Observational studies considering the empirical treatment of ESBL-related infections with BL/BLIs have infrequently included ICU patients [12] and more often involved urinary or biliary tract infection caused by *E. coli* species [51]. Although several studies suggested no difference in mortality [12, 51, 69], 2 publications raise the warning of a potential negative impact of BL/BLI when used in patients with ESBL-PE [19, 20].

Furthermore, when analyzing those publications with a focus on the use of alternatives to carbapenems for definitive therapy, a number of limitations hamper the interpretation of studies comparing BL/BLI to carbapenems.

Firstly most of these studies were not designed to compare different antibiotic strategies. Secondly, the authors did not take into account the severity of underlying diseases, delays to antimicrobial treatment and effectiveness source control, which are all major predictors of outcome [70]. Thirdly, patients included differed largely across studies, with regard to sources of bacteremia, species involved and type of beta-lactamases; moreover, various antibiotics and different daily doses administered were included in the “alternative” group.

Fourthly, most of the studies included infections related to ESBL-producing *Escherichia coli* and did not account for the impact of MICs and pharmacodynamics data. The impact of MIC seems to be crucial for therapeutic efficacy when using alternatives to carbapenems as the cornerstone of treatment. Several studies [12] emphasize the risk of treatment failure when using a BL/BLI or third-generation cephalosporin for therapy of infection with isolates having MICs higher than the breakpoints. Indeed, as suggested by a recent pharmacological study, the efficacy of BL/BLI in the treatment of ESBL-related infections is related to the concentration reached in the plasma and at the site of infection [71]. However, as demonstrated by several authors [72] the probability of attaining therapeutic drug levels in ICU patients is low and variable depending on the antibiotic considered and dosing strategies [35]. Also it seems important to have MIC for piperacillin–tazobactam before using this class of antibiotic. Considering several problems related to piperacillin–tazobactam gradient tests and differences noted between gradient tests and broth microdilution, it is recommended now to use broth microdilution.

There are now enough published data on the pk/pd of piperacillin/tazobactam to recommend the use of high daily doses and prolonged infusion ICU patients and in all cases of difficult to treat pathogens such as ESBL-PE.

The ecological consequences of a given antibiotic class depend on the amount of drug reaching the different microbiota. The net result depends on both the antibiotic concentrations achieved and the susceptibility of bacterial species in the microbiota. All antibiotics alter the composition, diversity and density of the microbiota and select for antibiotic resistance [73]. The “ecological consequences,” however, may differ according to the antibiotic used. Increasing consumption of carbapenems raises concerns on the spread of carbapenem-resistant Enterobacteriaceae and specifically carbapenemase-producing Enterobacteriaceae (CPE) [74]. Also, there are some discrepancies between the first published studies [75, 76] and the more recent ones [6] regarding the ecological effect of carbapenems. There is a significant correlation between carbapenem consumption and rates of *Pseudomona*s *aeruginosa* resistance to imipenem and meropenem [6, 53]. However, this mechanism of resistance is not due to the effect of antibiotics on the microbiota, but the consequence of chromosomal mutation. Earlier human studies [77] and animal models [78] suggested a limited impact on the microbiota of this class of antibiotics. However, whatever the antibiotic used, selective antibiotic pressure is an important determinant of emergence and dissemination of antibiotic resistance [61, 62], and the increasing use of carbapenems will necessarily be associated with the increase in multidrug-resistant organisms [65]. Our review underlines the fact that the administration of several other antibiotics can also be associated with the emergence of carbapenem-resistant organisms [60–64, 79–92]. Nevertheless, the heterogeneity of studies makes their comparison difficult. Indeed, all these studies are subject to several limitations, including inadequate adjustment for important confounding variables, control group selection, extent of prior antibiotic exposure and measurements of resistance outcomes.

One of the limitations of our study lies in the fact that we did not mentioned the two recent BL/BLIs approved by FDA and EMA, ceftolozane–tazobactam and ceftazidime–avibactam which are active in vitro against ESBL-producing Enterobacteriaceae. Several recent studies highlighted the in vitro efficacy of these two antibiotics on ESBL-producing Enterobacteriaceae [67, 93]. Also clinical data are scarce. Indeed for nosocomial pneumonia, a phase III study (MK-7625A-008) is currently leaded using ceftolozane–tazobactam.

A definitive answer to the question addressed in this review would need a randomized study conducted in ICU, including severe infections related to ESBL-PE. Cases should be selected according to the results of antibiotic susceptibility tests, and the trial should compare carbapenems to BL/BLI as definitive therapy. Pending such a trial, piperacillin–tazobactam should be used with caution for treatment of ESBL-PE-related infections. In ICU patients, empirical use should be avoided, and definitive therapy should be reserved to patients in clinical stable condition, after microbial documentation and results of susceptibility tests, together with adapting the administered dose and modalities of infusion to the MIC of the infecting microorganism in order to reach pharmacological targets.

## Abbreviations

ADE: antimicrobial de-escalation
BL/BLI: beta-lactam/beta-lactamase inhibitor
CLSI: Clinical and Laboratory Standards Institute
CRB: carbapenem-resistant bacteria
CRE: carbapenem-resistant Enterobacteriaceae
EMA: European Medicine Agency
ESAC: European Surveillance of Antimicrobial Consumption
EUCAST: European Committee on Antimicrobial Susceptibility Testing
FDA: Food and Drug Administration
ICU: intensive care unit
KPC: *Klebsiella*-producing carbapenemase
MIC: minimal inhibitory concentration
OR: odds ratio
PD: pharmacodynamic
PK: pharmacokinetic
TDM: therapeutic drug monitoring
TID: three times in a day
